# Supplementary material for: Two-Dimensional Iron Phosphorus Trisulfide as a High-Capacity Cathode for Lithium Primary Battery
Source: Molecules. 2023 Jan 5;28(2):537. doi: 10.3390/molecules28020537 (PMC9865732; doi:10.3390/molecules28020537)
Supplement: Supplementary file 1 [file molecules-28-00537-s001.zip › molecules-2087527-supplementary.pdf]

Electronic Supplementary Information

Two-dimensional Iron Phosphorus Trisulfide as a High-Capacity Cathode for Lithium Primary Battery

Syama Lenus,[a,b] Pallavi Thakur,[b] Sai Smruti Samantaray,[b] Tharangattu N. Narayanan,[b], and Zhengfei Dai [a*]*

List of Content:

Section S1—$FePS_3$ structure synthesis and preparation along with Digital Images

Section S2—Scanning electron microscopy (SEM)

Section S3—Energy-dispersive X-ray spectroscopy (EDS)

Section S4—Vibration sample magnetometry (VSM)

Section S5 —Thermogravimetric Analysis (TGA)

Section S6—X-ray photoelectron spectroscopy (XPS)

Section S7—Electrochemical cell specifications of $FePS_3$

Section S8—Cyclic Voltametric graph of $FePS_3$

Section S9—Cross-sectional SEM of electrode discharged at different stages

Section S10—Cross-sectional EDS of fully discharged electrode

Section S11—Full cell analysis

Section S12—Comparison Table

Section S13—Cost Analysis

Section S1—$FePS_3$ synthesis procedure

Preparation of NaCl Template: First, 2 M of NaCl aqueous solution (14.025 g of NaCl dissolved in 50 mL of water) was added into 200 mL ethanol and continuously stirred for 2 hours. The white NaCl precipitate was collected by filtration and washed with ethanol.

Synthesis of $FePS_3$ Nanosheets: The $FePS_3$ nanosheets were obtained by a facile NaCl-templated method. A total weight of 0.5 g of iron chloride ($FeCl_3.6H_2O$) was first dissolved into 50 mL of ethanol, followed by the addition of 5 g of as-prepared NaCl microcrystals. This solution mixture was then stirred at 50 °C until the whole solvent was evaporated completely. Thereafter, 0.5 g of phosphorus and 1.75 g of sulfur were added to the above iron-chloride-coated NaCl microcrystals. This mixture was then well-ground in a mortar. The resulting powder mixture placed in a ceramic crucible was then transferred into a heating furnace. The heating furnace consist of a closed system with no atmospheric air contact towards the material. Nitrogen gas was continuously passed through the annealing tube during the entire experiment and even before the experiment to remove the trapped oxygen. The nitrogen gas flow rate was maintained at 85 sccm.

The mixture was heated at a ramping rate of 2 °C min^{-1} until the temperature reached 310 °C in nitrogen atmosphere, and it was maintained at the same temperature for 5 hours. The temperature was further increased at a ramping rate of 3 °C min^{-1} to 500 °C, maintained for 3 hours. Once the annealing was completed, the temperature was brought down to 260 °C at a ramping rate of 3 °C min^{-1}, maintained for 2 hours, and then allowed to reach room temperature to obtain the final product. After the furnace treatment, the color changed from dark red to black, indicating the formation of the product. The formed product was washed vigorously with DI water. The final yield was dried at 60 °C for 6 hours.

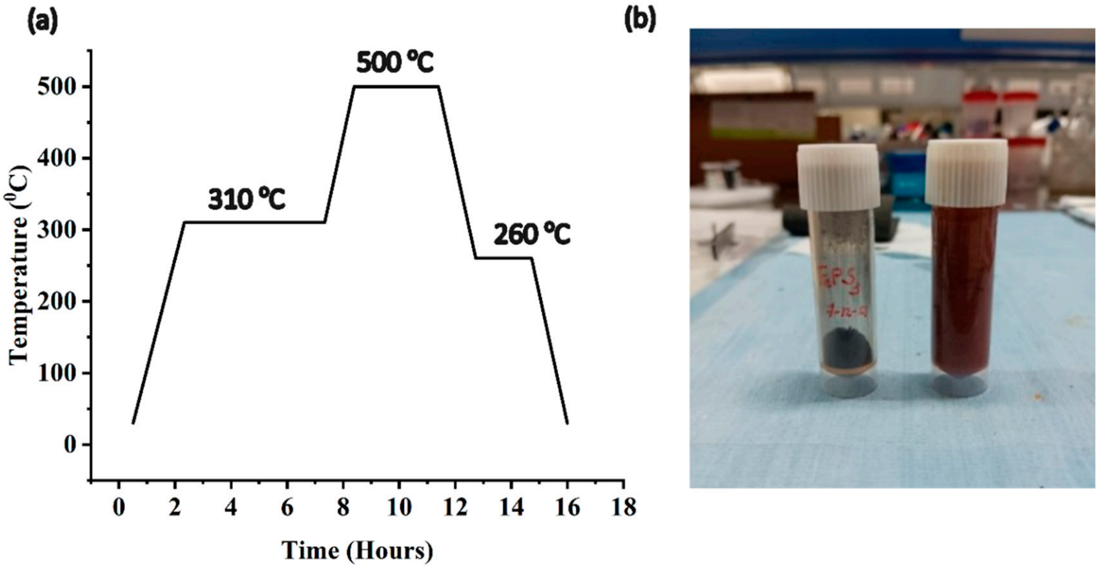

Figure S1. (a) Temperature chart of the synthesis procedure; (b) digital snapshot of the $FeCl_3$-coated NaCl ground with sulfur and phosphorus powder (right) and $FePS_3$ powder (left).

Section S2—Scanning electron microscopy (SEM)

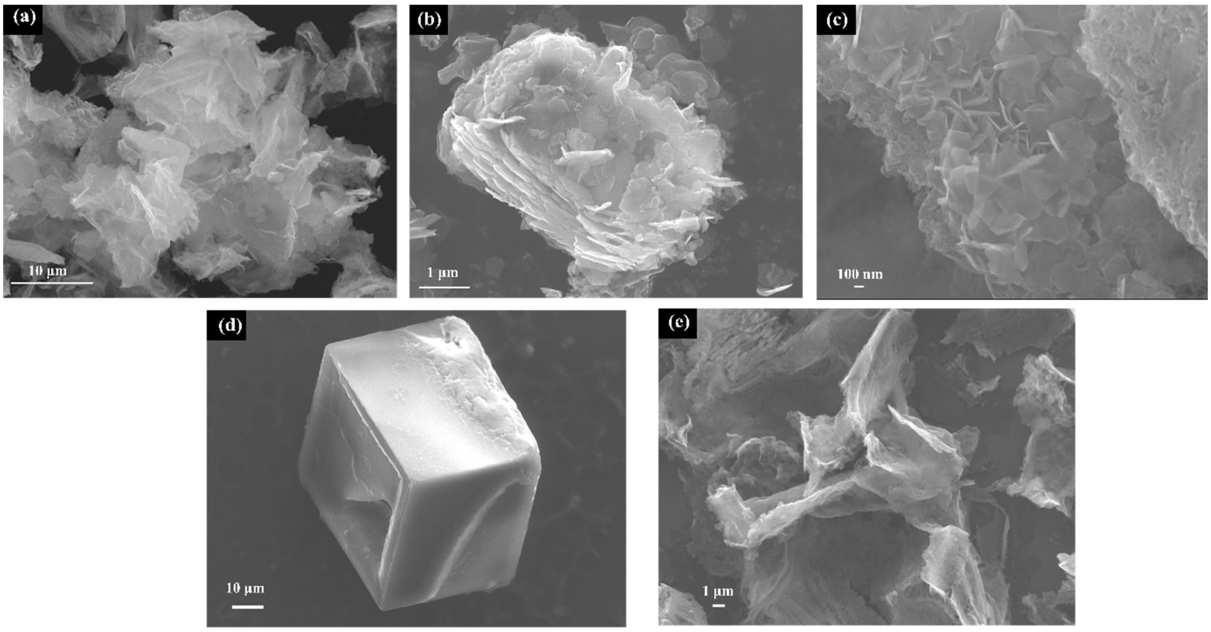

Figure S2. SEM images of $FePS_3$ at different magnifications: (a) at 10 µm, (b) at 1 µm, and (c) at 100 nm. The morphology of $FePS_3$ is identified as nanosheets in low magnification, which are formed from small nanoflakes, as can be seen at high magnifications. SEM images of different sizes of NaCl template and corresponding synthesized $FePS_3$. (d) NaCl template of size around 10 µm and (e) corresponding $FePS_3$ nanosheets.

Section S3—Energy-dispersive X-ray spectroscopy (EDS)

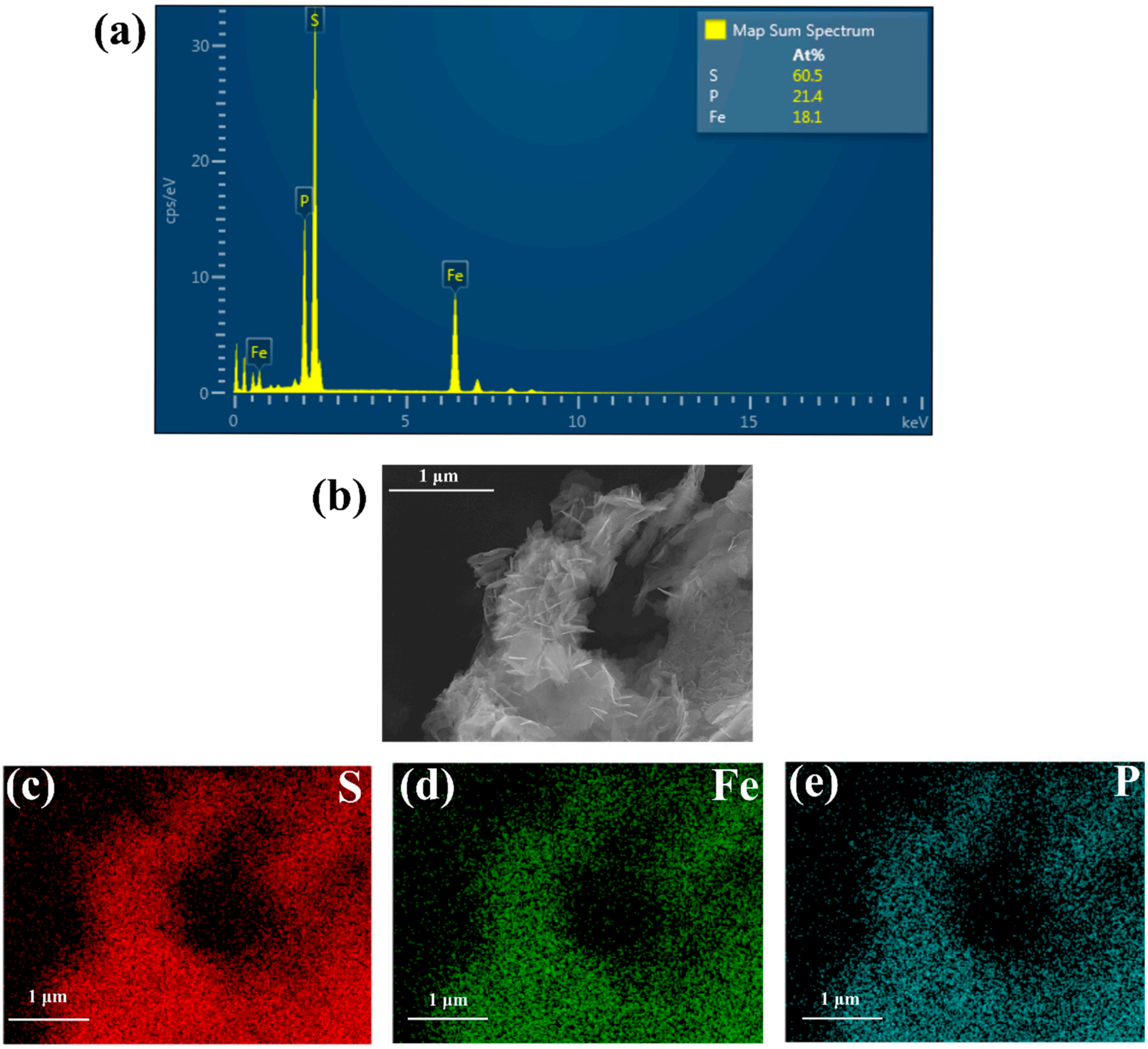

Figure S3. (a) EDS Spectra of $FePS_3$ at 10 µm; (b) electronic image of EDS maps. (c) Sulfur (S) in red color, (d) iron (Fe) in green color, and (e) phosphorus (P) in blue color.

Section S4—Vibrating sample magnetometry (VSM)

The magnetic measurements were performed using vibrating sample magnetometry. The study explains the degree of magnetization with respect to temperature at a constant magnetic field. The χ Vs T curves after field cooling (FC) and zero field cooling (ZFC) on pristine $FePS_3$ nanoflakes at an applied field of 100 Oe is depicted in Figure 4c. During the experiment, the sample was cooled from 300 to 50 K. The susceptibility of the sample was understood from FC and ZFC data. The susceptibility of synthesized $FePS_3$ was found to increase with a decrease in the temperature until its Neel temperature at 123 K. After the Neel temperature, the material shows a rapid decrease in magnetic susceptibility, showcasing the antiferromagnetic nature of $FePS_3$.

Section S5—Thermogravimetric Analysis (TGA)

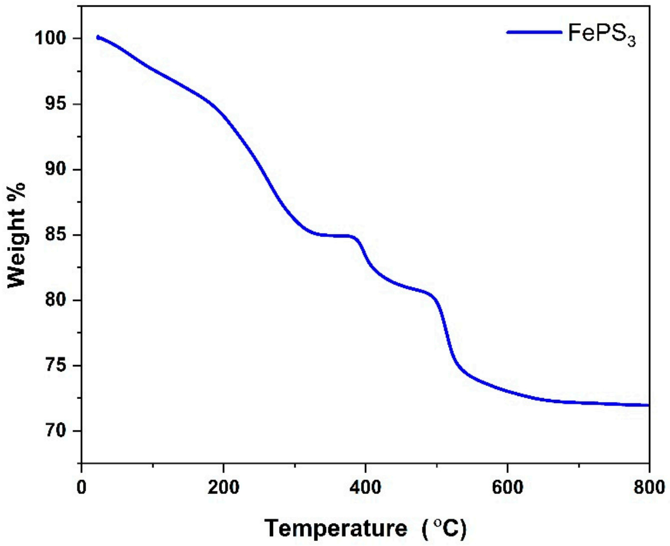

Figure S5. TGA thermogram of $FePS_3$ in the temperature range of 0 to 800 °C.

Thermogravimetric analysis (TGA) scrutinizes the thermal properties, particularly the decomposition temperature, of $FePS_3$ material. It is worth pointing out that the initial dip in the TGA is particularly because of the trapped water in the final step (washing) of the synthesis, which was eliminated up to 200 °C. Further, the second dip in the thermogram near 380 °C is due to the presence of FeS_x in the sample, while the major dip at around 520 °C is due to the dissociation of $FePS_3$.

Section S6—X-ray photoelectron spectroscopy (XPS)

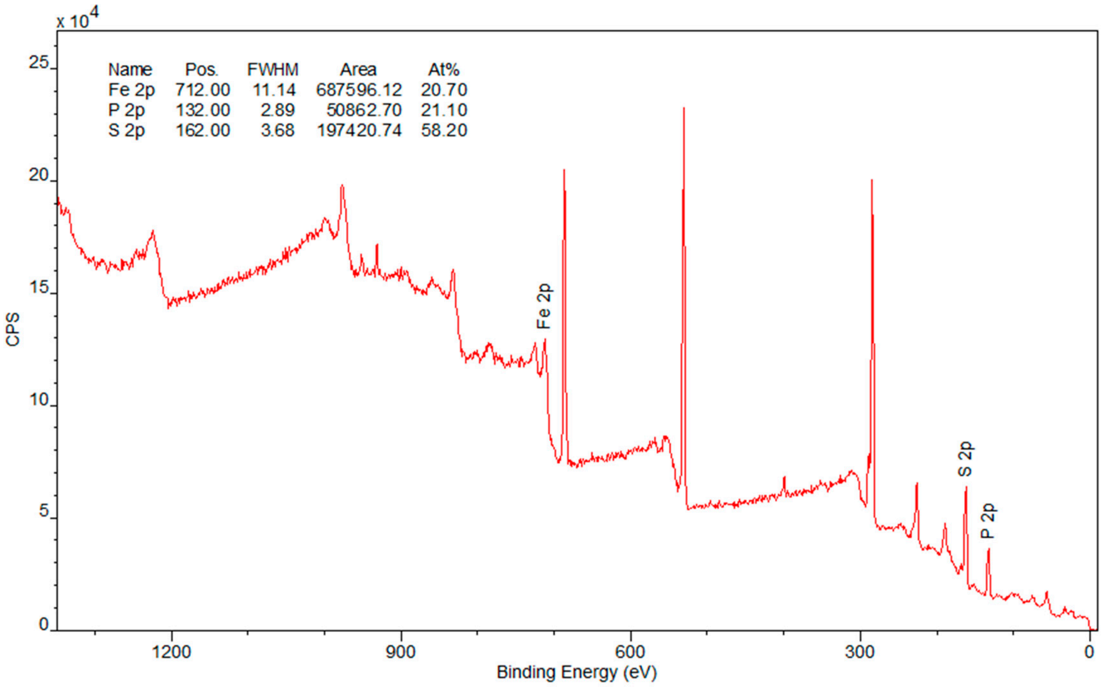

Figure S6.1. Survey spectra of pristine $FePS_3$.

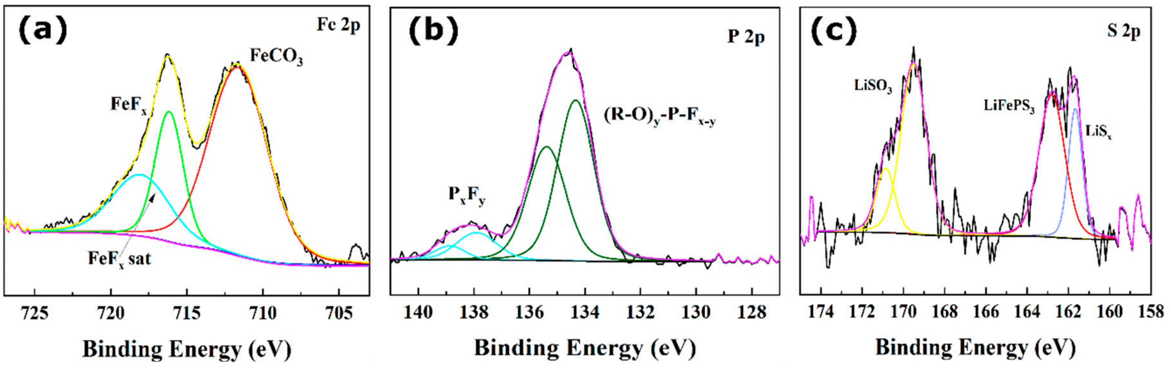

Figure S6.2. XPS analysis of fully discharged FePS3 electrode. (a) Decomposed product @Fe, (b) decomposed product @P, and (c) decomposed product @S.

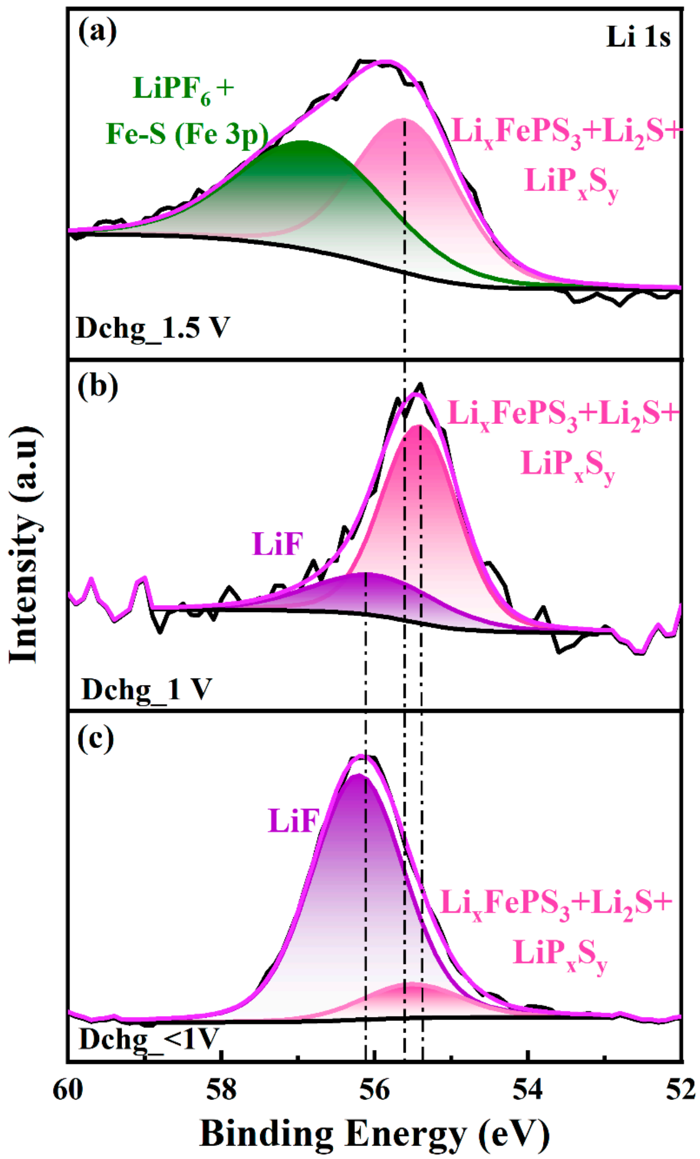

Figure S6.3. High-resolution spectra of Li 1s. $FePS_3$ electrode discharge (a) until 1.5 V, (b) 1 V, and (c) less than 1 V. *$LiPF_6$ is the electrolyte residue when electrode is taken out of the cell.*

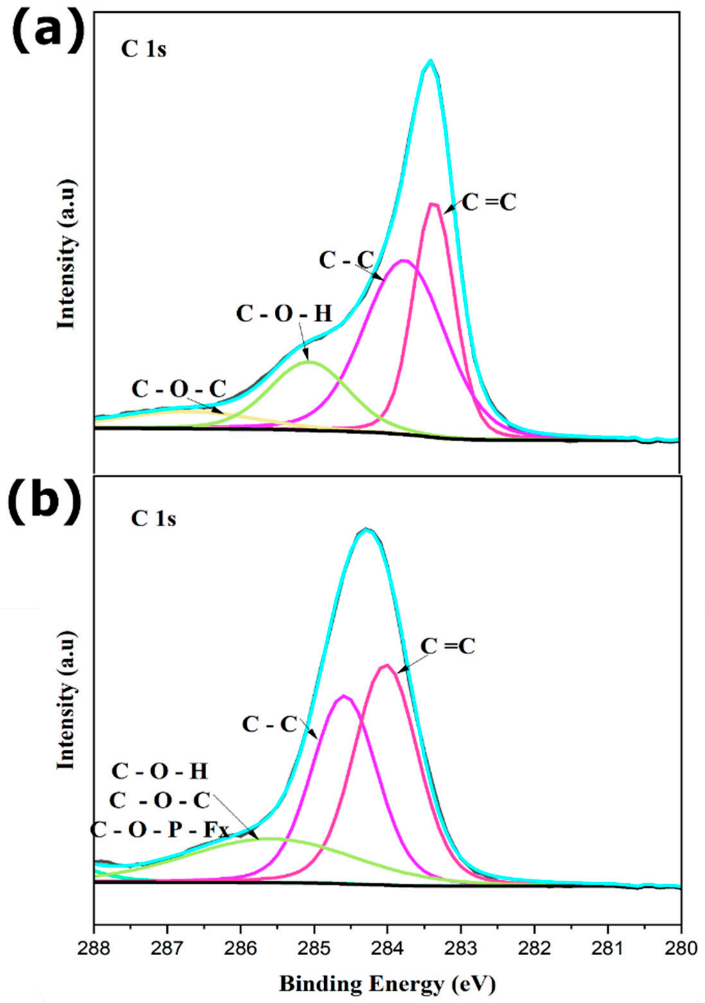

Figure S6.4 High-resolution spectra of C 1s. (a) Pristine $FePS_3$; (b) fully discharged $FePS_3$.

As shown in Figure S6.5, the XPS investigation at the initial stage of synthesis of $FePS_3$ (at 310 °C) for the photoemission lines of Fe 2p, P 2p, and S 2p were recorded. The high-resolution Fe 2p XPS spectrum of the sample shows the presence of Fe_xS_y moieties peaks at 713.5 eV (FeS), 709.7eV ($Fe_{III}S$), and 708.29 eV (FeS_2) (Figure S6.5a). It can be noted that the Fe 2p binding energy (BE) peak is not observed at 712.02 eV compared to the pristine $FePS_3$ peak at 712.0 eV (Figure 3a), indicating that the formation of $FePS_3$ has not started at the initial stage (310 °C). Similarly, the high-resolution P 2p XPS spectrum of the sample also supports the above-mentioned results. The P 2p spectrum shows the presence of P_xS_y units at 134.72 eV (P_4S_{10}), 132.56 eV (P-S-P), and 131.76 eV (P_4S_3) (Figure S6.5b). Furthermore, in accordance with the

above, both FeS_2 and P-S-P units were found at the high-resolution S 2p XPS spectrum (Figure S6.5c). The binding energy peak at 164.92 eV has both P-S-P and S moieties overlapping each other. Similarly, the binding energy peak at 162.2 eV indicates the presence of FeS_2 and P=S units overlapping each other. The binding energy peak at 163.6 eV confirms the presence of the P-S-P unit of P_4S_{10}.

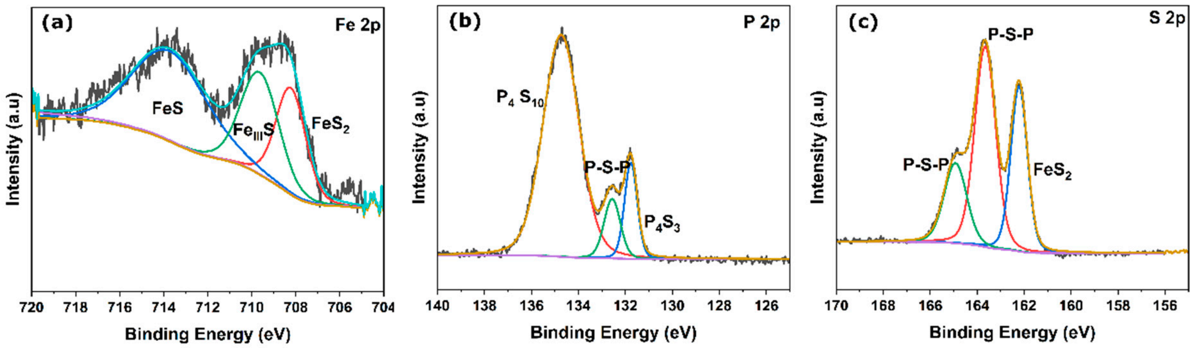

Figure S6.5. High-resolution spectra at initial stage of synthesis of $FePS_3$ (at 310 ^{o}C). (a) Fe 2p, (b) P 2p, and (c) S 2p.

Section S7—Coin cell testing specifications

Table S1. Coin cell testing specifications used in sample preparation for the characterizations.

Coin Cell Specifications	Cell type CR2016
**Anode active material**	$FePS_3$—80 wt.%, conductive carbon—wt.10%
**Binder**	PVDF wt.10%
**Cathode current collector**	Copper foil
**Counter electrode**	Li Foil (thickness: 1mm, diameter: 15.4 mm)
**Separator**	Celgard 2325
**Electrolyte**	1 M $LiPF_6$ in DMC
**Electrolyte amount**	0.25 mL
**Coin cell setup**	0.5 mm thick spacer and one spring.
**Active material loading**	0.9 to 1.9 mg
**Applied voltage range**	0.01 to 3.00 V
**Applied current density**	10 to 1000 mA/g

Section S8—Cyclic Voltametric graph of $FePS_3$.

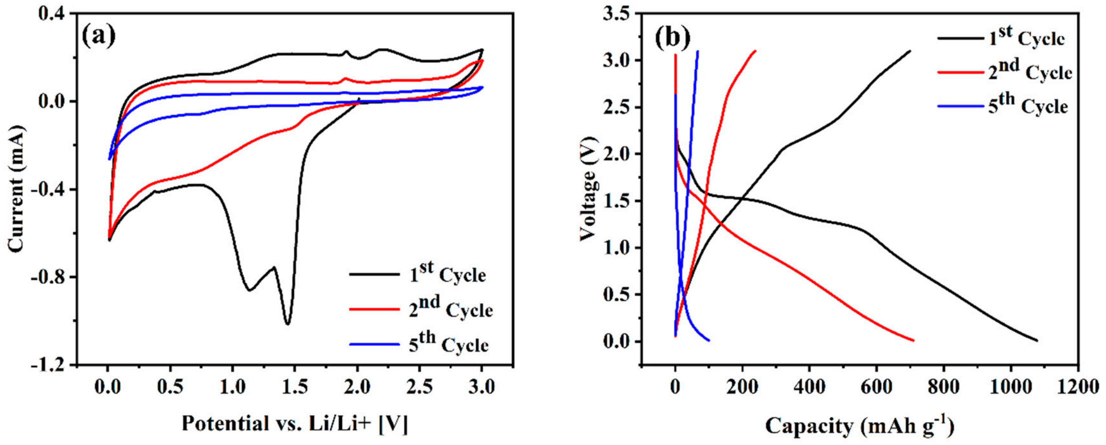

Figure S8. (a) Cyclic voltametric graph of $FePS_3$ for five cycles at a scan rate of 0.1 mVs^{-1}. (b) Galvanostatic charge discharge tests of $FePS_3$ as a secondary battery at a current density of 100 mA g^{-1}. As can be seen in Figure S7a, the current is reduced even after five cycles. Similarly, Figure S7b (ESI) shows the $FePS_3$ anode material tested as a secondary battery. Both these data indicate that the $FePS_3$ in the voltage window between 0.01–3.0 V failed to retain capacity after the first cycle.

Section S9—Cross-sectional SEM of electrode discharged at different stages

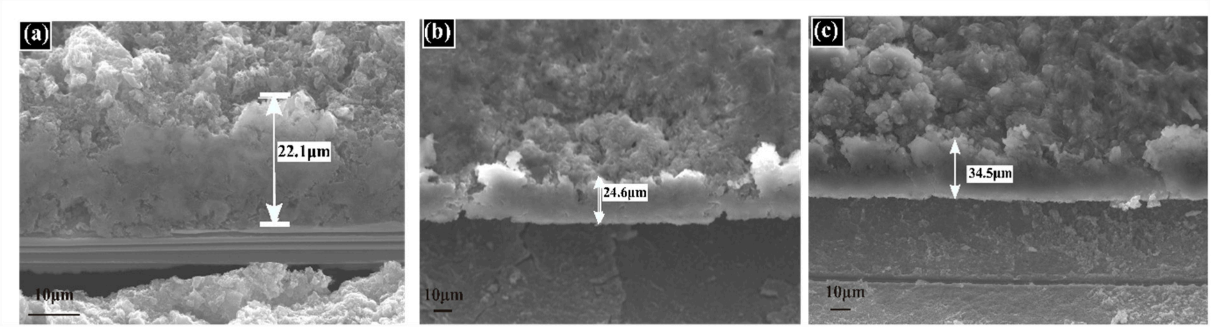

Figure S9. SEM images of cross section of electrode discharge at different voltages. (a) Uncycled electrode (b) discharged until 1.5 V, (c) discharged until 0.5 V, (d) discharged until .01 V.

Section S10—Cross-sectional EDS of fully discharged $FePS_3$ electrode

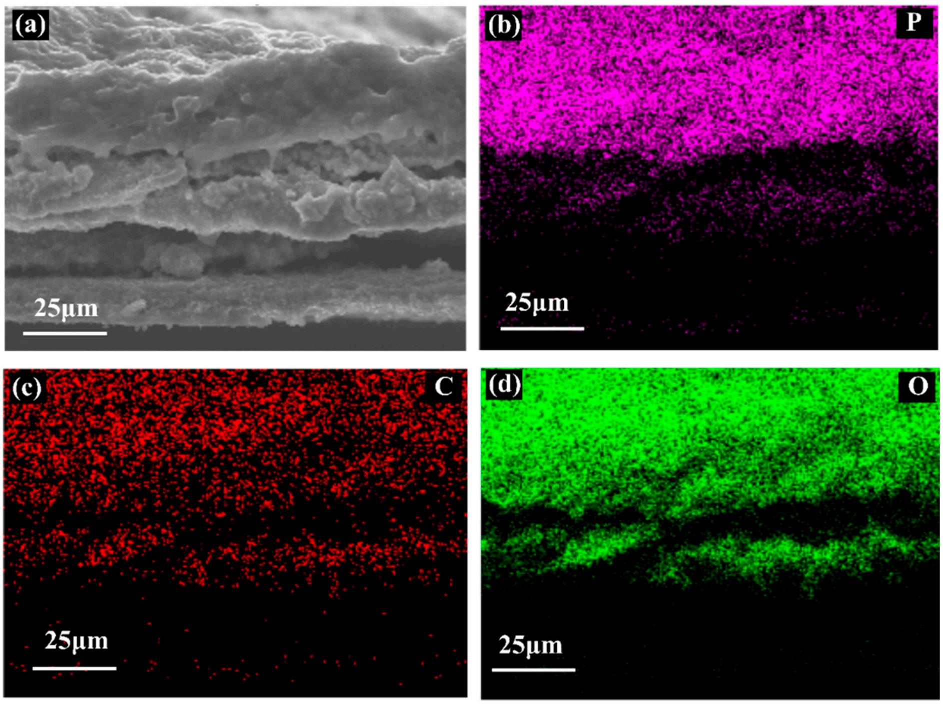

Figure S10. (a) Cross-sectional EDS maps of decomposed elements: (b) phosphorus (in violet frame), (c) carbon (in red frame), and (d) oxygen (in green frame).

Section S11—Full cell analysis

Full cell analysis was conducted using lithiated graphite as anode and $FePS_3$ as cathode. Lithiated graphite was prepared prior to the full cell experiment. Graphite electrode was constructed using graphite, conductive carbon, and PVDF in 8:1:1 ratio and dispersed in NMP. The slurry was coated on a battery-graded copper foil using doctor blade technique and dried at 80 °C for 8 hours. A CR2016 coin cell was assembled using lithium as anode and graphite electrode as cathode. The coin cell was discharged at a current density of 10 mA/g. The above cell was then opened to obtain the lithiated graphite electrode.

Further, a new CR2016 coin cell was assembled using lithiated graphite as anode and $FePS_3$ as cathode. This cell was discharged at a current density of 10 mA/g. A discharge capacity of 1131 mAh g^{-1} (Figure S11 (a)) was obtained for $FePS_3$, which was limited by the low lithium content in the graphite, while the discharge capacity was obtained w.r.t., the full amount is 247 mAh g^{-1} (Figure S11b).

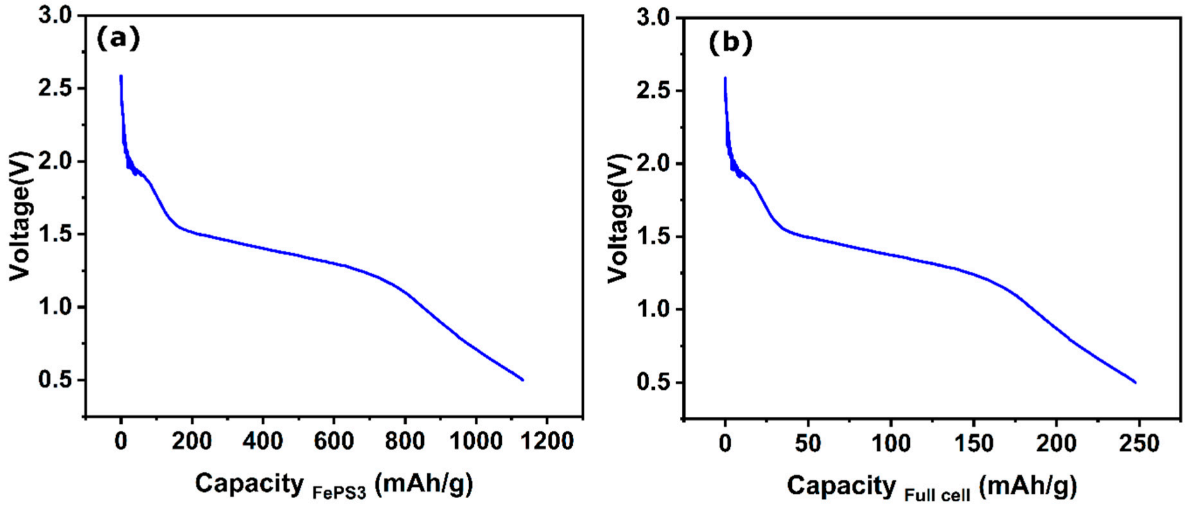

Figure S11. Capacity vs. voltage plot of galvanostatic full discharge of Li vs. $FePS_3$ coin cells considering the weight of (a) $FePS_3$ only and (b) $FePS_3$+graphite.

Section S12—Comparison Table

Table S2. Comparison table compares as-synthesized $FePS_3$ with other previously reported lithium primary battery systems.

Material	Capacity (mAh g^{-1})	Energy Density (Wh g^{-1})	Current Density	Voltage Range (V)	Ref
**$FePS_3$**	**1940**	**2400**	**10 mA g^{-1}**	**1.0–3.0**	**This work**
**$FePS_3$**	1160	1770	-	1.0–3.0	[20]
**Fluorographene**	520		1 C	1.5–3.0	[44]
**CFx**	753	800	1 C	1.5–3.0	[45]
**α-CuV_2O_6**	514	-	20 mA g^{-1}	3.5–2.0	[12]
**AQ-DME-5%FEC**	575	1300	20 mA g^{-1}	1.5–3.0	[3]
**FGS-x**	828.4	830.7	1 C	1.5–3.0	[48]
**FGCNT-x**	798.8	2006.6	10 mA g^{-1}	1.5–3.0	[10]
**Li/CF_x**	767	1600	10 mA g^{-1}	1.0–3.5	[6]
**CuF_2/CF_x-1.0**	670.45	-	10 mA g^{-1}	1.5–3.5	[9]
**Li/Cu2S**	309	497	0.25 mA cm^{-2}	0.5–2.0	[52]
**Li/CdS**	363	428	0.25 mA cm^{-2}	0.5–2.0	[52]
**Li/PbS**	209	289	0.25 mA cm^{-2}	0.5–2.0	[52]
**Li/CFx**	837.4	2004.5	10 mA g^{-1}	1.5–3.5	[53]
**Li/CF_x**	910.5	722.8	0.05 C	1.5–3.0	[54]
**CF_x**	921	2210	10 mA g^{-1}	1.5–3.0	[8]
**IFDO**	652	1392	-	1.5–3.0	[56]
**FG/S-1**	1024	2341	0.05 A g^{-1}	1.5–3.0	[57]
**HT-CF**	735	1867	C/30	1.0–4.0	[58]
**LT-$CF_{1.0}$**	619	1950	C/30	1.0–4.0	[58]
**LT-$CF_{0.9}$**	611	1967	C/30	1.0–4.0	[58]
**LT-$CF_{0.8}$**	530	1728	C/30	1.0–4.0	[58]
**$CF_{0.84}$**	785	1147	40 mA g^{-1}	1.5–3.5	[59]
**PTh/CFx**	715	1707	0.05 C	1.5–3.0	[60]
**FG**	632	1485	10 mA g^{-1}	1.5–3.5	[61]
**$BaFeO_4$**	310	-	-	0.5–3.5	[62]
**$SrFeO_4$**	380	-	-	0.5–3.5	[62]
**K_2FeO_4**	400	-	-	0.5–3.5	[62]
**Li_2FeO_4**	600	-	-	0.5–3.5	[62]
**PPy/SVO**	297	-	C/25	1.5-3.5	[63]

AQ and activated by the presence of an electrolyte additive, fluoroethylene carbonate (FEC).

FGS-x—fluorinated graphene microspheres

(FGCNT-x)—fluorinated graphitized CNTs

IFDO—indeno[3,2-b]fluorene-6,12-dione

FG/S-1—fluorinated graphene (FG)/sulfur hybrid

HT-CF—high temperature

LT-CF—low temperature

Section S13—Cost Analysis

Table S3. The estimated cost analysis of a single coin cell based on different cathodes.

Materials	Cost USD
**$FeCl_3$**	1.85 /Kg
**Red phosphorus**	5.47 /Kg
**Sulfur**	0.3 /Kg
**NaCl**	20 /Kg
**Lithium foil**	1.9/Kg
**Electrolyte**	1.2 /Can
**Coin cell parts**	0.15 /piece

Cost analysis was performed considering lowest price precursors from the India Mart shopping site. The price for lithium foil and coin cell parts was estimated from the Made-in China shopping Site. The estimate was made for coin cell using 1 g of lithium foil. The rate in kilograms was converted to grams to estimate total price as USD 0.17, in which USD 0.15 is for coin cell assembling parts.
